# Small-molecule inhibitor of Bcl-2 (TW-37) suppresses growth and enhances cisplatin-induced apoptosis in ovarian cancer cells

**DOI:** 10.1186/s13048-015-0130-x

**Published:** 2015-02-20

**Authors:** Haixia Wang, Zhifeng Zhang, Xiuping Wei, Ruizhen Dai

**Affiliations:** Department of Blood Transfusion, The Affiliated Hospital of Weifang Medical College, Weifang, China; Department of Blood Transfusion, TaiAn Central Hospital, TaiAn, China; Department of Blood Transfusion,people’s hospital of Weifang traditional Chinese medicine, Weifang, China; Department of Joint Surgery, The Affiliated Hospital of Weifang Medical College, Weifang, China

**Keywords:** Ovarian cancer, Cisplain, Bcl-2, TW-37

## Abstract

**Background:**

Bcl-2 plays a major role in the pathobiology and drug resistance of ovarian cancer, and inhibition of bcl-2 was useful for OC therapy. It has previously reported that TW-37, a small-molecule inhibitor of Bcl-2 family proteins, inhibited cell growth and induced apoptosis in many cancer cells. In the present study,we investigate the effect of TW-37 or / and in combination with cisplain on several ovarian cancer (OC) cell lines with high bcl-2 expression.

**Methods:**

The bcl-2 mRNA and protein expression, and the cisplain (DDP) sensitivity of OC cell lines SKOV3, OVCAR3, OV-90 and 3AO and SKOV3^DDP^ were determined by Quantitative real-time RT-PCR,Western blot, and 3-(4,5-dimethylthiazol-2-yl)-2,5-diphenyltetrazolium bromide and fluorescence-activated cell sorting (MTT) assays. The effects of TW-37 alone or combined with cisplain on growth and apoptosis in bcl-2 overexpressed OVCAR3, OV-90 and SKOV3^DDP^ cells was detected by MTT,clonogenic assay, ELISA and terminal deoxynucleotidyl transferase-mediated nick end labeling (TUNEL) assay.

**Results:**

The cell lines SKOV3 and 3AO were sensitive, whereas OVCAR3, OV-90 and SKOV3^DDP^ were resistant to cisplain. Significant positive correlation was observed between basal bcl-2 mRNA and protein and cisplain sensitivity. Cisplain treatment did not activate bcl-2 in vitro. Treatment with TW-37 inhibited bcl-2 expression in bcl-2 overexpressed OVCAR3, OV-90 and SKOV3^DDP^ cells , and inhibited growth and induced apoptosis ,and increased cisplain killing of the bcl-2 overexpressed cells in a does and time-dependant manner in vitro.

**Conclusion:**

Bcl-2 level positively correlated with sensitivity to cisplain. Treatment with TW-37 was effective alone and in combination with cisplain in bcl-2 overexpressed OC cell lines in vitro. Thus, TW-37 may be a useful therapeutic agent for OCs.

## Background

Ovarian carcinoma (OC) continues to be the leading cause of death due to gynecologic malignancy in the world because it is usually diagnosed in the advanced stage of the disease [1,2]. The standard treatment for epithelial ovarian cancer remains surgical debulking and chemotherapy with a platinum and taxane agent. Although many patients with disseminated tumors respond initially to standard combinations of surgical and cytotoxic therapy, nearly 90% of them develop recurrence [3].

Cisplatin (DDP) and its analogues are first-line chemotherapeutic agents for the treatment of human ovarian cancer [4,5]. Cisplatin promotes its cytotoxicity by forming DNA-protein cross-links, DNA mono-adducts, and intrastrand DNA cross-links, which all trigger apoptosis [6,7]. In ovarian cancer, the majority of tumours acquire drug resistance. Response rates to first-line platinum-based therapy are more than 80%, but most patients with advanced disease will finally relapse and die because of acquired drug resistance [8]. The mechanisms involved in cisplatin resistance are not yet fully understood.

Ovarian cancer like many other tumors has been shown to overexpress the Bcl-2 and/or its family members [9-12]. Tumors expressing high levels of Bcl-2, Mcl-1, or Bcl-XL, are often found to be resistant to chemotherapeutic agents or radiation therapy [13]. Therefore, novel avenues by which Bcl-2 could be inactivated represent a promising strategy for the development of novel and selective anticancer therapies. The Bcl-2 family members are important proteins that regulate the program cell death in cancer cell lines. It includes both death antagonists such as Bcl-2, Bcl-XL and Mcl-1 as well as death agonists such as Bax, Bak, Bid and Bad [14]. An imbalance between antiapoptotic proteins (such as Bcl-2, Bcl-XL and Mcl-1) and proapoptotic proteins (such as Bax and Bcl-xs) is involved in the distinctive biological features of adenocarcinomas [15].In epithelial ovarian cancer, anti-apoptosis proteins Bcl-2, Bcl-XL and Mcl-1 are highly over-expressed [9-12]. Clinical data showed that the enhanced expression of Bcl-2 and Bcl-XL is related to a shorter patient survival, whereas the upregulation of Bax is associated with longer survival and these findings suggest that the modulation of apoptotic pathways might be one of the reasons why epithelial ovarian cancer shows only limited sensitivity to anticancer treatment [10-12]. Thus, blockade of Bcl-2 activity represents a novel and promising strategy for designing new class of anticancer drugs that can overcome the resistance of cancer cells to chemotherapy or radiation.

TW-37 is a potent small-molecule inhibitor of BCL-2, which attenuates BCL-2 activation and inhibits multiple BCL-2 family members including BCL-XL and MCL-1. It binds to the BCL-2 homology domain 3 (BH3) groove of BCL-2 preventing the heterodimerization of proapoptotic proteins (such as Bid, Bim, and Bad) with BCL-2 and subsequently allowing them to induce apoptosis [16]. Recent studies indicate TW-37 is able to inhibit the growth of a broad range of cancer cells, since it induces S-phase cell cycle arrest with regulation of several important cell cycle related genes, including p27, p57, E2F-1, cdc25A, CDK4, cyclin A, cyclin D1 and cyclin E [17,18].

Thus, in the present study, we investigated whether TW-37-induced inhibition of epithelial ovarian cancer growth could be attributed to Bcl-2 inactivation *in vitro*, and whether TW-37 increased the sensitivity of ovarian cancer cells to DDP.

## Materials and methods

### Cell culture

Ovarian cancer cell lines SKOV3, OVCAR3, OV-90 and 3AO were obtained from the American Type Culture Collection (ATCC; Shanghai, China). Cisplatin (DDP) resistant SKOV3 cell line (SKOV3^DDP^) was obtained from yiyeqi.cc (Shanghai, China). The cell lines were grown in RPMI 1640 (Invitrogen) supplemented with 10% FBS. SKOV3^DDP^ was dissolved in DMSO (Novaplus, Ben Venus Laboratories, Inc.) was added.

### Chemicals

Cisplatin (DDP, cis-diammine-dichloro-platinum II) and MTT [3-(4,5-dimethylthiazol-2-yl)-2,5- diphenyltetrazolium bromide] were purchased from Sigma-Aldrich (St. Louis, MO). Stock DDP solution was prepared in DMSO (330 mM), stored as aliquots at 20°C, and used within 2 weeks. DDP was further diluted in medium before adding to the cells. TW-37 was obtained from Selleck Chemicals Co. (Shanghai, China).

### Cytotoxicity assay

The microculture tetrazolium assay was used to measure cytotoxicity as described earlier [19]. Treatment consisted of continuous incubation with cisplatin (Pharmacochemie BV, Haarlem, the Netherlands) or WT-37 for 96 h. The mean IC50 ± s.d. was determined in three experiments, each performed in quadruplicate.

### Cell growth inhibition assay by MTT assay

Cells were seeded in 96-well plates at 5 × 10^3^ per well and treated with varied concentrations (250 nmol/L, 500 nmol/L, and 750 nmol/L) of TW-37 for different times (24 hs, 48 hs and 72 hs). After treatment, cell densities were determined by the MTT assay. The results were plotted as means ± SD of 3 separate experiments having four determinations per experiment for each experimental condition. In addition to the above assay, we have also done clonogenic assay for assessing the effects of treatment as shown below.

### Soft-agar colony assay

Cells were plated (50,000–100,000 per well) in a six-well plate and incubated overnight at 37°C. After 72-h exposure to various concentrations of TW-37, then the cells were plated in soft-agar at 37°C. The colonies in the soft agar were counted in all untreated and treated wells after 12 days as described before [20].

### Quantification of apoptosis by ELISA

The cell death detection ELISA (enzyme linked immunosorbent assay) kit was used for assessing apoptosis according to the manufacturer’s protocol. Briefly, cells were treated with TW-37 for different periods of time. After treatment, the cells were lysed and the cell lysates were overlaid and incubated in microtiter plate modules coated with antihistone antibody for detection of apoptosis.

### TUNEL assay

Cells were treated with TW-37 for 72 h, as described above. After treatment, cells were washed with cold PBS and fixed in ethanol for 1 h. The cells were then stained with 5 μg/mL Hoechst for 30 min and visualized under a fluorescence microscope. Bright condensed, punctuate, or granular nuclei were considered apoptotic. Moreover, terminal deoxynucleotidyltransferase-mediated nick end labeling (TUNEL) was assayed with a commercial apoptosis detection kit (Promega Corp.)[17].

### Western blot assay

Cells treated with different concentrations of cisplatin or or/and TW-37, sonicated three times, cleared by centrifugation at 10,000 × g for 10 minutes at 4°C, and immunoblotted using antibodies for Bcl-2 and β-actin. Data were expressed as relative fold expression overβ-actin.

### Quantitative real-time RT-PCR

Real-time RT-PCR for bcl-2 and housekeeping gene GAPDH was done using iScript One-Step RT-PCR kit with SYBR Green (Bio-Rad), according to the manufacturer's instructions.The primer sequences used were as follows: bcl-2 forward primer 5’-CTCCTGACGCTAAGAGCTTCG-3’ , reverse primer 5’-CCAGGCTGGAAGGGAAAGAC-3’;GAPDH forward primer 5’-CCTGGCACCCAGCACAAT-3’ , reverse primer 5’-GGGCCGGACTCGTCATCG-3’.

### Statistical analysis

All data are presented as the mean ± SD. The data were examined using analysis of variance (ANOVA) and the least significant differences method for multisample comparisons or Student’s t-test for two sample comparisons. P < 0.05 was considered as statistically significant.

## Results

### Bcl-2 is overexpressed in ovarian cancer cell lines

**Figure 1:**
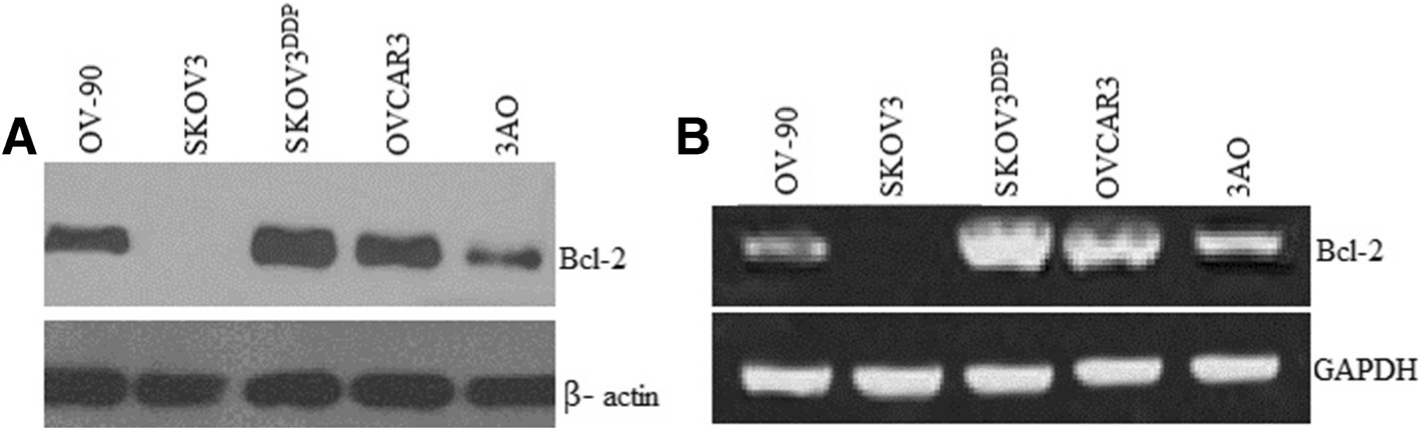
**Western blot and Q-RT-PCR assay for bcl-2 protein and mRNA in SKOV3, OVCAR3, OV-90,3AO and SKOV3** ^**DDP**^ **ovarian cancers cell lines.** bcl-2 protein and mRNA was highest in DDP resistant SKOV3 cell (SKOV3^DDP^).The experiments are repeated for three times. The representative figs were shown. **A**, Western blot; **B**, Q-RT-PCR.

Using total RNAs and proteins from SKOV3, OVCAR3, OV-90 , 3AO and SKOV3^DDP^ovarian cancers cell lines, we show using real-time RT-PCR and immunoblotting that all ovarian cancer cell lines, except SKOV3 VO-90 and 3AO, express higher levels of bcl-2 mRNA and protein, respectively. SKOV3^DDP^ovarian cancers cell expressed the highest bcl-2 levels (Figure 1A and B).

### Ovarian cancer cell lines vary in resistance to DDP

**Figure 2:**
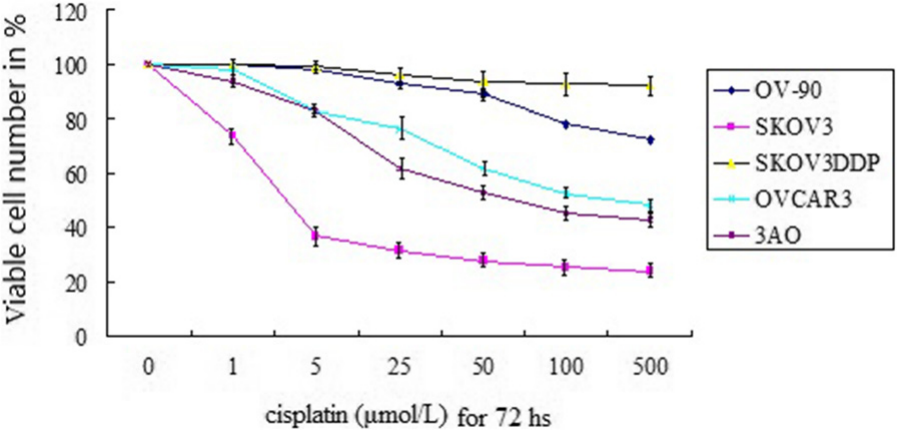
**Ovarian cancer cells have differing levels of native resistance to cisplatin.** Human OC cell lines SKOV3, OVCAR3, OV-90, 3AO and SKOV3^DDP^ were treated with increasing concentrations of cisplatin (0–500 umol/L) for 72 h. The viabilities indicated on the y axis were determined by MTT assays and normalized to control. Data shown are means ± SE for n = 3 independent experiments.

SKOV3, OVCAR3, OV-90, 3AO and SKOV3^DDP^ were treated with different concentrations of cisplatin (1, 5,10,25,50 ,100 and 500 μmol/L) for 72 hours and the number of surviving cells was analyzed. Whereas the cisplatin LD50 was around 10 μmol/L −50 μmol/L for 3AO and SKOV3 cell lines, which has very low bcl-2 levels. The cisplatin LD50 was around 100 μmol/L for OVCAR3 and OV-90, and > 500 μmol/L for SKOV3^DDP^ cell lines, which has highest bcl-2 levels (Figure 2).The same sensitivities were obtained when the effects of cisplatin were analyzed on apoptosis using TUNEL and ELISA (data not shown). Therefore, we hypothesized that bcl-2 mRNA or protein levels would predict chemoresistance. These data supported the classification of the SKOV3 and 3AO cell lines as cisplatin-sensitive, and OVCAR3, OV-90, SKOV3^DDP^ cell lines as cisplatin-resistance.

### Cisplatin treatment did not affect bcl-2 mRNA and protein level

Although basal bcl-2 level reflect cisplain sensitivity, it remained possible that responses to stress may be different in sensitive versus resistant cells. Therefore, we wished to evaluate whether bcl-2 in response to treatment with cisplain could predict sensitivity. SKOV3, OVCAR3, OV-90, 3AO and SKOV3^DDP^ were stimulated with various concentrations of cisplain (1 μmol/L-500 μmol/L) for 24 hours in vitro and bcl-2 mRNA and protein were measured using Q-PCR and western blot assay.Cisplain (1 μmol/L-500 μmol/L) treatment for 24 hours had no significant effect on bcl-2 level in any of the cells analyzed (Data not shown).

### TW-37-induced cell growth inhibition by MTT assay

**Figure 3:**
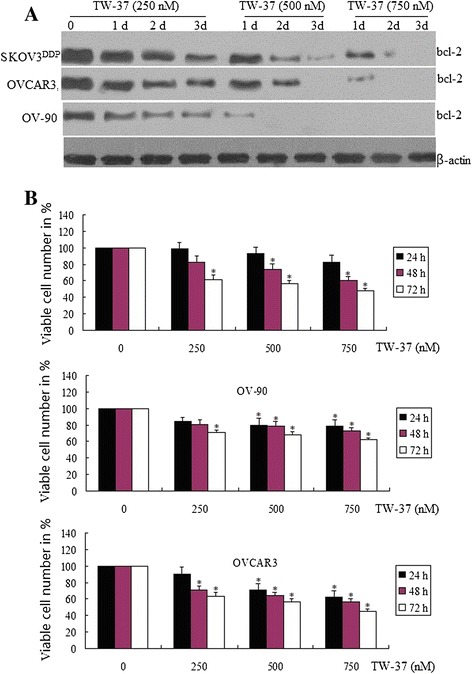
**Effect of TW-37 on bcl-2 expression and growth in OVCAR3, OV-90 and SKOV3** ^**DDP**^ **cells.** The treatment of OVCAR3, OV-90 and SKOV3^DDP^ cells for 1–3 days with 250, 500 and 750 nM of TW-37. **(A)** Dose and time responses of TW-37 on bcl-2 inhibition in OVCAR3, OV-90 and SKOV3^DDP^ cells by western blot assay. **(B)** Dose and time responses of TW-37 on growth of OVCAR3, OV-90 and SKOV3^DDP^ cells. Data shown are means ± SE for n = 3 independent experiments. Vs control, ^*^ *P* < 0.05.

We next examined the growth inhibitory effects of TW-37 using the MTT assay in 3 OC cell lines such as OVCAR3, OV-90 and SKOV3^DDP^. The reason for choosing these 3 OC cell lines was due to the fact that these cell lines showed higher expression of Bcl-2. The treatment of OC cells for 1–3 days with 250, 500 and 750 nM of TW-37,the bcl-2 was significantly inhibited (Figure 3A), and the cell growth inhibition in a dose- and time-dependent manner in all 3 OC cell lines (Figure 3B) In addition, we have also tested the effects of treatment on cell viability by clonogenic assay as shown below.

### TW-37-induced cell growth inhibition by clonogenic assay

**Figure 4:**
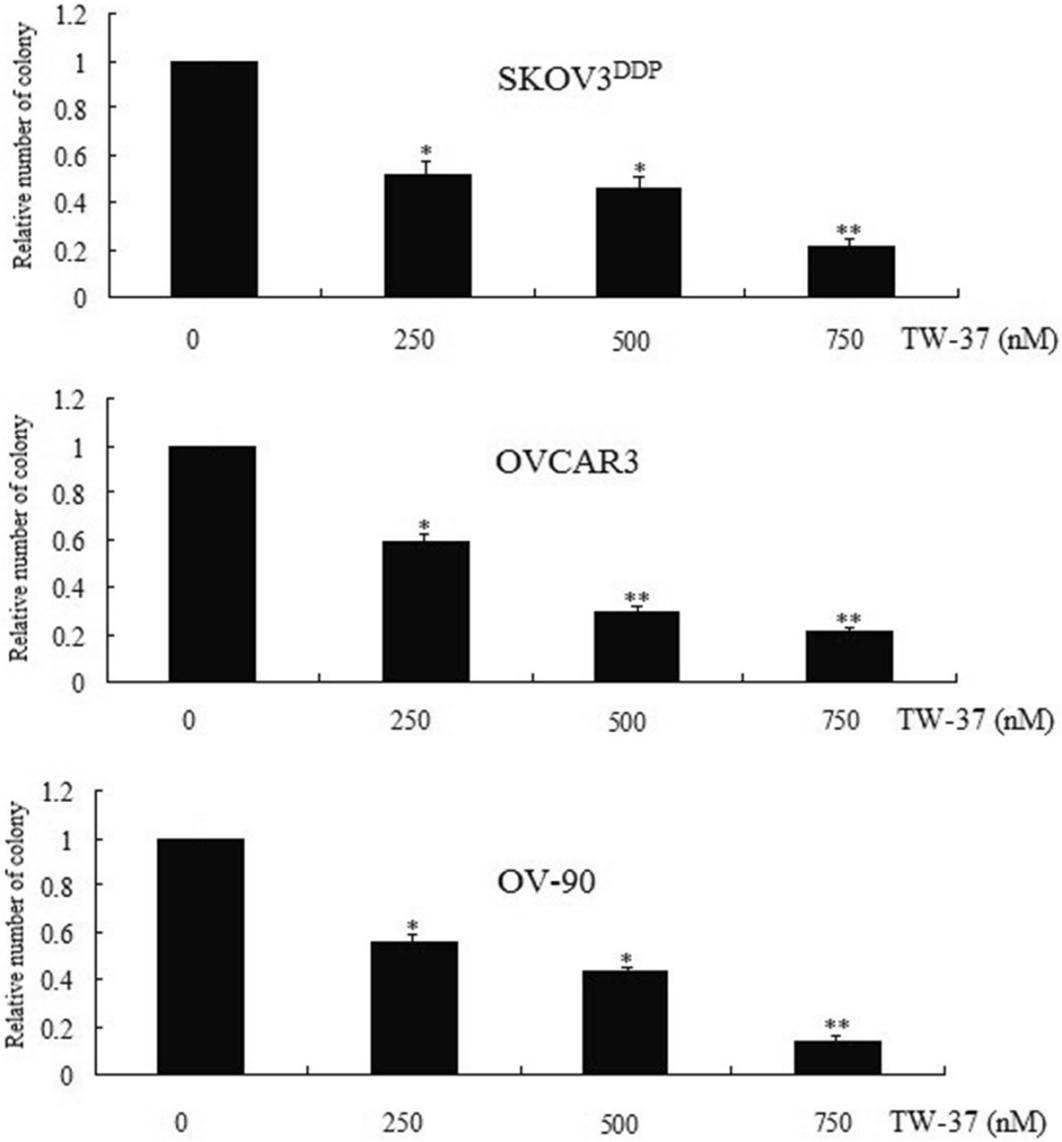
**Effect of TW-37 on Soft-agar colony formation in OVCAR3, OV-90 and SKOV3** ^**DDP**^ **cells.** OVCAR3, OV-90 and SKOV3^DDP^ cells treated with 250, 500 and 750 nM of TW-37 for 72 hs were evaluated by the Soft-agar colony assay. The colonies in the soft agar were counted in all untreated and treated wells after 12 days. There was a significant reduction in the colony formation in OVCAR3, OV-90 and SKOV3^DDP^ cells treated with TW-37 compared with control cells ( the control was as 1). The experiments are repeated for three times. *P* values represent comparisons between cells treated by TW-37 and control using the paired *t* test. Vs control, ^*^ *P* < 0.05; ^**^ *P* < 0.01.

To determine the effect of TW-37 on cell growth, cells were treated with TW-37 and assessed for cell viability by clonogenic assay. OVCAR3, OV-90 and SKOV3^DDP^ cells were treated for 3 days with 250, 500, and 750 nmol/L of TW-37. TW-37 resulted in a significant inhibition of colony formation of OVCAR3, OV-90 and SKOV3^DDP^ cells when compared with control (Figure 4). Overall, the results from clonogenic assay was consistent with the MTT data as shown in Figure 3B, suggesting that TW-37 inhibited cell growth in OVCAR3, OV-90 and SKOV3^DDP^ cells. To confirm cell growth inhibition, we have also conducted the apoptosis assay induced by TW-37.

### TW-37 induced apoptosis by TUNEL and ELISA assay

**Figure 5:**
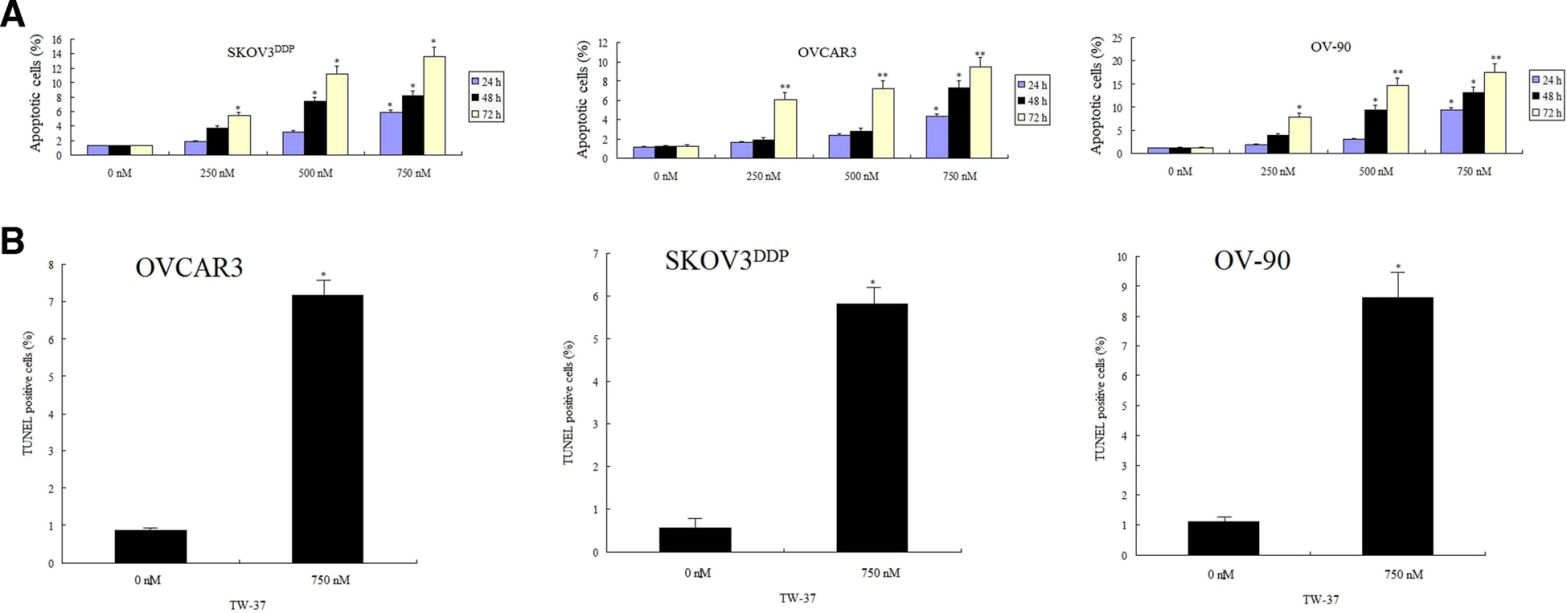
**Effect of TW-37 on OC cell apoptotic death.** **A**, OVCAR3, OV-90 and SKOV3^DDP^ cells were treated with 250, 500 and 750 nM of TW-37 for 24–72 hr. Apoptosis was measured by ELISA. **B**, TUNEL was performed in OVCAR3, OV-90 and SKOV3^DDP^ cells treated with 750 nM TW-37 for 72 h using TUNEL assay. The experiments are repeated for three times. Columns, mean; bars, SD. *P < 0.05, **P < 0.01, compared with the control.

OVCAR3, OV-90 and SKOV3^DDP^ cells were treated with 250, 500 and 750 nM of TW-37 for 24–72 hr. After treatment, the degree of apoptosis was measured by ELISA assay. We found that TW-37 induced apoptosis in a dose- and time-dependent manner (Figure 5A). To confirm this result, we also used TUNEL methods to detect apoptosis: OVCAR3, OV-90 and SKOV3^DDP^ cells were treated with 750 nM of TW-37 for 72 h. TUNEL assay also showed that TW-37 induced apoptosis in OVCAR3, OV-90 and SKOV3^DDP^ cells (Figure 5B).

### TW-37 potentiates growth inhibition induced by DDP in OVCAR3, OV-90 and SKOV3^DDP^ cells

**Figure 6:**
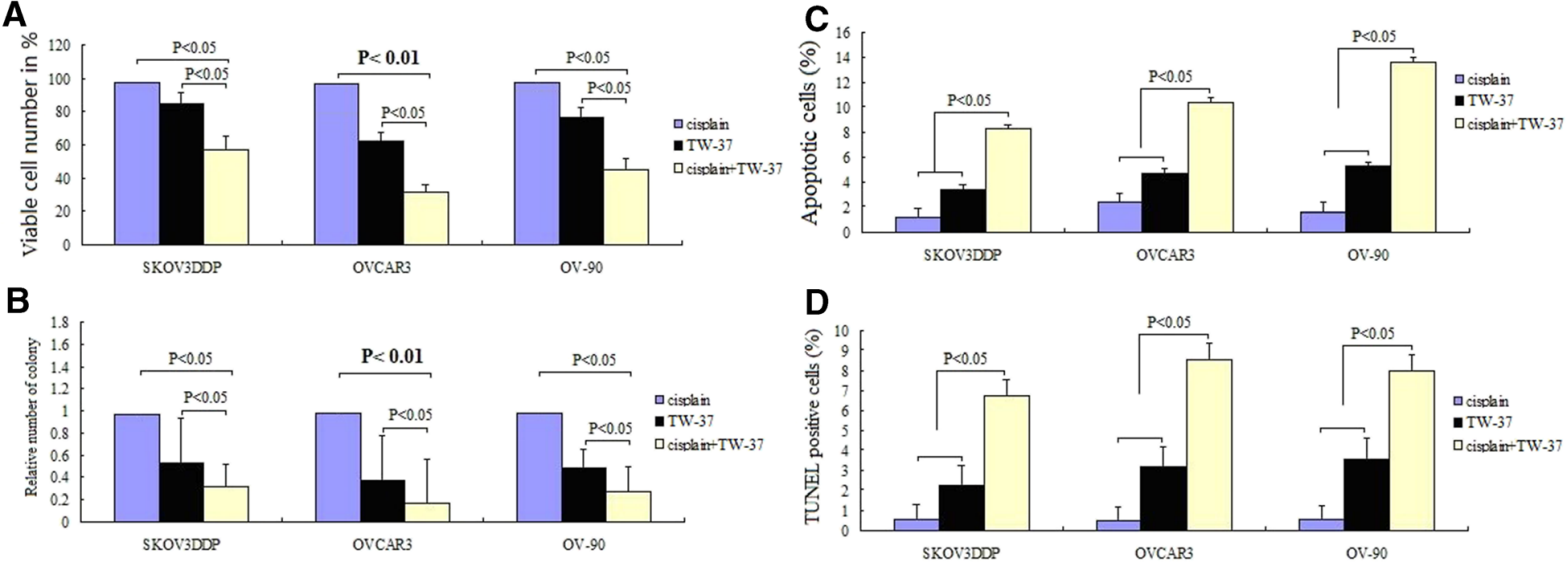
**Effect of TW-37 on cisplain induced growth inhibition and apoptosis on OVCAR3, OV-90 and SKOV3** ^**DDP**^ **cells.** OVCAR3, OV-90 and SKOV3^DDP^ cells treated with 500 nM of TW-37 combined with 50 μmol/L of cisplain for 72 hs were evaluated by MTT **(A)**, Soft-agar colony assay **(B)**, ELISA **(C)** and TUNEL assay **(D)**. The experiments are repeated for three times.

We assessed the effect of pretreatment and cotreatment of a combination of 500 nM of TW-37 and 50 μmol/L of cisplain on cell viability by MTT assay. Viable cells were evaluated at 72 hs posttreatment. Pretreatment with TW-37 for 24 hours followed by treatment with cisplain resulted in significant cell growth inhibition in the three cell types investigated (Figure 6A). We next assessed the effect of pretreatment and cotreatment of a combination of 500 nM of TW-37 and 50 μmol/L of cisplain on cell viability by clonogenic assay.TW-37 resulted in a significant inhibition of colony formation of OVCAR3, OV-90 and SKOV3^DDP^ cells when compared with control (Figure 6B).

### TW-37 sensitizes OVCAR3, OV-90 and SKOV3^DDP^ cells to apoptosis induced by DDP

Relative to single agents, pretreatment of 500 nM of TW-37 followed by 50 μmol/L of cisplain treatment induced much more apoptosis in the three cell lines as shown by ELISA (Figure 6C) as well as TUNEL analysis (Figure 6D). These results are consistent with cell growth inhibition studies by MTT, suggesting that the loss of viable cells by TW-37 and cisplain is partly due to the induction of an apoptotic cell death mechanism.

## Discussion

The Bcl-2 family of proteins plays critical roles in human cancers, including OC. The activation of Bcl-2 has been shown to enhance tumor growth and inhibition of apoptosis. The overexpression of Bcl-2 family proteins in OC may also play important roles in resistance to a wide spectrum of chemotherapeutic agents [21].

Bcl-2 appears to be a compelling target for treatment of patients with OC. Therefore, identification of an inhibitor targeting Bcl-2 family of proteins is likely to provide a therapeutic benefit for OC. Our current data show that TW-37 not only inhibits cell growth and induces apoptotic cell death of OC cells, but also sensitized OC cells to cisplain treatment.

TW-37, a recently developed small-molecule inhibitor of Bcl-2, is capable of antagonizing the function of pan-Bcl-2 family and thereby may have greater therapeutic potential as an entirely new class of antitumor agent. It has found that TW-37 inhibits the growth of a variety of cancer cells [17,22,23]. Here, we investigated the mechanism by which TW-37 elicits its biological effects on OC cells. In this study, we used bcl-2 overexpressed OVCAR3 and OV-90 cell line, and cisplain resistant SKOV3 cell line (SKOV3^DDP^). All the three cell lines have high expression of Bcl-2 mRNA and protein. We found that TW-37 was capable of inducing significant growth inhibition in the cells as detected by the MTT assay and the clonogenic assay. Moreover, TW-37 also induced apoptotic cell death in all the cell lines, suggesting that blocking Bcl-2 is sufficient to trigger apoptosis in OC cells overexpressing the bcl-2 protein.

Whether a high level of bcl-2 gene is directly associated with resistance to DDP treatment in human OC cells remains controversial. Our study did show a positive relationship between cisplatin sensitivity and the level of bcl-2 in the OC cell line. In the 3AO and SKOV3 cell lines, which has low bcl-2 mRNA and protein level, the cisplatin LD50 was around 10 μmol/L-50 μmol/L; in the OVCAR3 and OV-90 cell lines, which has high bcl-2 mRNA and protein level, the cisplatin LD50 was over 100 μmol/L; In the SKOV3^DDP^ cell line, which has the highest bcl-2 levels, the cisplatin LD50 was over 500 μmol/L. Because cisplain treatment had no significant effect on bcl-2 expression in mRNA and protein (data not shown) in any of the OC cells analyzed, we therefore suggested that endogenous Bcl-2 level did correlate with sensitivity to cisplain in OC cell line in vitro.

Western blotting revealed that TW-37 block Bcl-2 in all the three high blcl-2 expressed OC cells in a concentration dependent and time-dependent manner. The reduction of bcl-2 levels correlated with the enhancement of cisplain-induced apoptosis and growth inhibition. In this study, we found that TW-37 pretreatment enhanced significant tumor cell killing compared with either agents alone. In the cisplain resistant SKOV3 cell line ( SKOV3^DDP^), TW-37 treatment could significantly restore the chemotherapy sensitivity to cisplain. Inhibition of cell growth was also correlated with apoptotic cell death. We are currently performing experiments to evaluate in more depth possible mechanistic explanations for these results. Nevertheless, these results guided our decision to start both drugs at the same time in our in vivo studies.

## Conclusions

We presented experimental evidence, that endogenous Bcl-2 level was positively correlated with sensitivity to cisplain in OC cell line. It also strongly supports the antitumor effects of TW-37 in OC in vitro. It is possible to enhance chemosensitivity of OC cells by pretreatment with TW-37 leading to apoptotic cell death. However, further mechanistic studies could be useful to fully support our strategy for the treatment of patients with OC.

## Abbreviations

TW-37: Small-molecule inhibitor of Bcl-2
MTT: 3-(4,5-dimethylthiazol-2-yl)-2,5-diphenyltetrazolium bromide and fluorescence-activated cell sorting assays
TUNEL: Terminal deoxynucleotidyl transferase-mediated nick end labeling assay
ELISA: Enzyme linked immunosorbent assay
